# Education and technology synergy in environmental sensing

**DOI:** 10.1098/rsta.2024.0281

**Published:** 2025-07-31

**Authors:** John Selker, Chester Udell

**Affiliations:** ^1^ Dewpartment of Biological and Ecological Engineering, Oregon State University, Corvallis, OR, USA

**Keywords:** instrumentation, education, environmental sensing, undergraduate

## Abstract

Advancing understanding of climate change and ecological vulnerability requires new talent and new tools. The Oregon State University Openly Published Environmental Sensing (OPEnS) Laboratory, founded in 2015, works to enpower talented undergraduate students with challenging opportunities as they address their personal values through innovation in Earth sensing systems. OPEnS’ overarching goal is to develop sensing technologies addressing critical environmental challenges, advancing both scientific discovery and technological innovation. OPEnS inventions leverage novel microelectronic sensors, three-dimensional printed components, embedded microprocessors, parametric system design, wireless telemetry and cloud-based data delivery. A key area for development is what we refer to as ‘Born FAIR’ data systems (sensor-embedded code so data are Findable, Accessible, Interoperable and Reusable) where sensor data are fully contextualized by metadata collected at installation. OPEnS interweaves technological progress with educational opportunities seeking to develop the next generation of sensor systems and their engineers. Despite openly published designs, most users of OPEnS technology do not wish to make their own gear, and thus OPEnS is increasingly engaging industrial partners. OPEnS has shown that direct connections between scientific clients, teams of engineering students, industrial collaborators, national labs and international partners advance scientific discovery and technological innovation.

This article is part of the Royal Society Science+ meeting issue ‘Hydrology in the 21st century: challenges in science, to policy and practice’.

## 1. Empowered designers

In the literature and in product catalogues, we see environmental sensing systems employing 1980s technologies. While ‘tried and true’ methods are essential to scientific advancement, ‘working as in the past’ may not serve the joint explosions of *need* for global environmental sensing and *opportunity* to implement modern technological capabilities (e.g. three-dimensional design, embedded microprocessors, sensors, edge computing, telemetry and interoperable data systems). An unsolved riddle is how to bring about the transformational opportunities presented by this suite of technologies: almost no single lab has the expertise to incorporate these capacities, while almost every team studying Earth needs these tools [1]. Discovery is being held back. Earth science needs a *community effort* devoted to the resolution of pressing measurement technologies. At Oregon State University, we have prototyped a facility and organizational structure. This paper describes the unexpected opportunities that were revealed and calls on the scientific community to join the effort.

Bouncing along in a van full of graduate students from the San Francisco American Geophysical Union (AGU) meeting in December 2014, Dr. Clement Roques and Selker wrote up the first grant proposal to start the Openly Published Environmental Sensing (OPEnS) lab. Our goal was to make sensors advancing scientific discovery of Earth systems by combining the power of new micro-sensors with open-source micro-computers, 3D printing and wireless communication with funding of just $50 k yr^−1^. We budgeted for two undergraduates, some basic electronics gear, a laser cutter and a single 3D printer.

A key feature of the OPEnS proposal was that it would be an undergraduate-led design facility. This was opportunistic, in that we had seen a key population of secondary school students become deeply engaged in the ‘maker movement’, where they learn combined mechanical, electronic and computer skills as they build robots and other projects. We have observed a dramatic increase since our inception of incoming autodidactic students driven by curiosity and a passion to create, likely related to the expansion of secondary school Science, Technology, Engineering and Mathematics (STEM) programmes. These students are bringing maker skills to university, seeking to fill out the context for their passions. We saw that the OPEnS lab could provide them with a hands-on complement to their courses, wherein they can apply both their experiential and academic training to solve problems that align with their values. We posited that by entrusting the students with the greatest possible sense of ownership, the lab would provide them with the greatest possible learning experience. Students would test and develop their own abilities to plan, execute and continuously improve an engineering programme. Integrating their personal experience, values, academic training and agency created a uniquely exciting environment for highly motivated students. The lab managerial structure consists of the lab principal investigator (Selker), a half-time director (Udell), a half-time lab manager and other professional staff, who act as design mentors rather than bosses of student teams.

We set clear and ambitious expectations: each project should be designed to provide a significant improvement in environmental sensing capability for a client and should be publishable in the peer-reviewed literature. While lowering costs is a primary objective for many makers, we prioritize innovations that could lead to scientific discovery. However, our designs illustrate that innovation and low cost are not mutually exclusive.

A challenge to OPEnS lab is that undergraduates move on to graduate school or their careers—generating high staff turnover. This demands meticulous engineering practices, keeping work fully documented on GitHub and continually being ready to pass the baton. The COVID pandemic made OPEnS adopt a more hands-on process of project development, including 360-degree reviews with each team on a weekly basis. The fact that over half of the completed projects in OPEnS have resulted in undergraduate-led peer-reviewed publications reflects the impact of fully engaged mentoring [2–13].

Our foremost objective was to facilitate scientific advancement through new sensor systems, but an emergent feature of OPEnS was that engaging students in critical scientific tasks created a potent educational opportunity. Here’s one of our unexpected takeaways: when you want to address a hypothesis, get a graduate student, but when you want to facilitate discovery by solving a technical problem, hire undergraduate engineering students. The undergraduates have a predilection to pursue well-defined problems with an end clearly in sight, whereas graduate students generally seek to advance knowledge through open-ended exploration. Undergraduates have a burning desire to translate their book learning into real-world impact, and they bring and advance cutting-edge technical skills. Most OPEnS students stay with the lab for over 2 years, including summers. They benefit technically and educationally from collaborating. In OPEnS, we thought our main output would be new tools, until we saw what the 200 graduates of the OPEnS lab went on to do professionally. In building an energized instrument development laboratory, we had accidentally hit upon an educational jackpot.

Hands-on education is critical in engineering education, and it is becoming harder to come by. From Leonardo da Vinci to the machines of the Industrial Revolution, learning to build was a self-taught process of exploration with mentored apprenticeships. Selker’s own grandfather had only a sixth-grade education but went on to invent the automotive shock absorber, among others [14]. Engineering education has become increasingly virtual with hands-on experiences becoming scarce (e.g. [15], pp. 66, 67, 69, 72, 105, 107, 173). STEM education is at a crossroads [16]. We know that hands-on problem-based learning in multi-disciplinary teams working on complex systems is the most fruitful strategy. Most universities have not built the space for this approach in their curriculum [15,17]. While the iterative design process and structured developmental cycles that take a product from concept to completion can be taught in formal educational settings, these projects are often limited to the scope of the classroom and the length of the academic term. Too many great designs are left forgotten in the desk drawer after the final grade is issued. OPEnS addresses this issue for hundreds of students. Students translate the needs of real-world clients and issues into user and system specifications. These undergo refinement through iterative in-lab prototyping and testing, typically spanning over four years and three ‘generations’ of student engineers. Students learn to produce calibration, system validation, quality assurance and quality control (QA/QC) criteria and test their designs to prove functionality before anything goes to the field. Outdoor testing reveals the key impacts of the environment on functionality so the students directly see the results of their decisions. Testing starts in the lab, then to the immediate environment of the university and finally to the client’s sites. The challenges of nature (including, among others, floating logs, crushing snowpacks, grizzly bears and leaking rubber seals). These indelible experiences prepare students for the challenges they will face in their professional lives. The OPEnS lab is now having students evaluate their design’s maturity via the NASA Technology Readiness Levels approach [18]. This tool provides a validated framework for students to self-appraise their progress.

While giving a tour of OPEnS on a recent Sunday, we found five students in the lab working out the next generation of air quality monitoring systems (table 1, last line). Building on their success in installing 50 systems last summer, they were adding sensors for CO, CO_2_ and O_3_ as requested by the client. Majoring in electrical engineering, computer science, mechanical engineering and ecological engineering, they were addressing the real-world needs of researchers supporting farmers from California to Washington. Engineering decisions, bugs and their discovered solutions, as well as each line of code, are documented on GitHub so the next versions will grow efficiently from their work. This is an example of the value-driven, problem-based learning that undergraduate students need to be prepared to serve their world. This student-engineered air-quality sensing system is now being considered by the USGS to monitor volcanoes and globally to study urban air quality.

**Table 1.** Selected examples of OPEnS sensor systems now in use from a total of 28 deployed client-driven sensing systems for diverse Earth science applications. All designs and code are published openly on GitHub.

problem	solution	exemplar
*water bodies*. No tool for long-term autonomous collection of sequential eDNA samples.	*PolyWAG* [13,19] 24-sample eDNA sampler. Smartphone programmable; sequential isolation to 1 : 100 000 000: preserves samples. Used by NOAA, EPA, Universities.	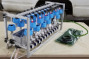
*forest, urban, agriculture*. Need temperature-stable, 0.5-micron accuracy, zero-friction dendrometer to measure soft and woody tissue.	*dendrometer* [20] two patents issued, one pending, improvement in accuracy, zero-friction, temperature-stable device. >100 in use and in licensing discussions with a US producer.	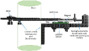
*climate, ag, ecology*. No rain gauges for extreme precipitation environments where maintenance is not feasible.	*evaporometer* [11]. Measures both rainfall and evaporation to 0.05 mm with no moving parts or parts needing cleaning. In licensing to a US producer.	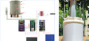
*hydrology*. No large-capacity isotopic samplers with low fractionation.	*OPEnS-Iso* [21] 3D print float-valve and aluminium-bag collection. Reduces fractionation 5×, costing 1/10 of leading brand.	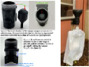
*ecosystems, ag*. No trapping method for non-hormonal mating insect species.	*Pied Piper* [12]. Edge-computed Fourier-spectra detect target mating call; acoustic response attracts and captures.	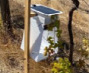
*hydrology, hazards*. No durable user-buildable multi-sonde for stream monitoring for science and education.	*SmartRock* [22,23]. Novel sample-and-hold measurement of electrical conductivity, temperature, pressure, dissolved oxygen and turbidity. In use in the USA, Europe and Taiwan.	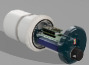
*climate, health, ag*. No available multi-parameter air quality sensor with 4G connectivity for forest fire impact, urban and volcano studies.	*WISP*. 4G telemetry user-selectable gas species plus standard 1, 2.5, 4.0, 10 m aerosol, VOC, NOx, temp, humidity. Deployed in CA, OR, WA.	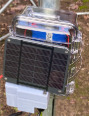
*outreach, co-design*. No socially or economically acceptable landslide monitoring system for Southeast Alaska.	*WeatherChimes* [11]. Co-designed through iterative hands-on workshops and field deployment activities with local science centres and high school training programmes. Tracks rainfall, groundwater depth, soil moisture, water temperature, air temperature, relative humidity and sunlight.	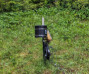

Students thrive on the excitement of being part of scientific discovery where they can apply cutting-edge technologies to critical environmental issues (e.g. [24]). What we saw that Sunday was representative: engagement in environmental monitoring allows students to *be agents of positive change in problems of burning concern*. These projects bring forth the most potent learning environment and provide a pipeline for the next generation of leaders in the global industry of environmental sensing [25].

Nine years after its establishment, the concept has been validated. OPEnS includes 30−40 undergraduates who lead multi-disciplinary teams (mechanical, electrical, computational, telemetric and biophysical) under the mentorship of graduate students, professors, the lab’s clients and industrial collaborators who seek to commercialize the new tools. OPEnS has had over 20 paying clients seeking solutions to scientific measurement needs, from improved precision in measuring the methane emissions from cows, to monitoring forest fire air quality impacts on wine grapes, to understanding the dynamics of plant stems under water stress. Undergraduate students have written 14 peer-reviewed articles (three in review), obtained two patents (three in review) and are currently negotiating licensing agreements with industrial partners. OPEnS tools are used by scientific teams across the US, Germany, France, Switzerland, Israel, Taiwan, Japan and Ghana. The success of OPEnS has far outgrown its foundations and has shown the remarkable pent-up demand for such innovation.

Beyond technology development, students learn the value of liberal education. Does a tree falling in a forest with no one there to hear it make a sound? Our sensors say yes. However, does a great sensor design that is never deployed make a difference to the world? No. Nor do otherwise well-trained engineers who cannot share their ideas. Professor Melanie Green, in *How Stories Connect and Persuade Us: Unleashing the Brain Power of Narrative,* says: ‘Solid information in any form is good, but that’s not necessarily enough. A vivid, emotional story can give that extra push to make it feel more real or more important. If you look at the times somebody’s beliefs have been changed, it’s often because of a story that hits them in the heart’ [26]. The OPEnS lab seeks to train its students to communicate their ideas and developments. All students take part in documentation on GitHub, and most are authors on one or more papers. Four to five students are funded each year to bring their instrumentation and present a poster to an audience of over 25 000 scientists, educators, policymakers, journalists and communicators from around the world at the AGU Annual Meeting. Translation is also essential at the sensors: in Southeast Alaska, OPEnS uses data sonification (turning data into music) and storytelling in hands-on sensor workshops co-designing environmental monitoring systems for coastal communities [27]. We are now developing a relationship with the theatre arts community at Oregon State so that our students can study and practice the basic art of storytelling. Udell, the OPEnS director, is the Artist in Resident at the Hatfield Marine Science Center for the 2025−2026 season, combining students, OPEnS technologies and creative practice to translate data and science concepts into impactful experiences.

## 2. Technological context

Climate change and ecological collapse will be central to the landscape of science for the foreseeable future. Environmental sensing requires precise instrumentation capable of maintaining function under harsh conditions. It is widely understood that the opportunities for developing new sensor systems have grown dramatically with the advent of ever more accurate, economical and low-power electronic sensors. However, several other transformative parallel developments amplify today’s opportunities:

— *Machine discoverable data*. Sensors should be launched with the metadata embedded based on globally supported standards of automated time-of-installation metadata collection that can be automatically discovered and incorporated into external research efforts (what we refer to as Born FAIR—see below).
— *Engage the internet*. Networks of sensors can deliver massive datasets, which now can be globally available and efficiently processed with cloud tools. Additionally, the internet creates opportunities for outreach and storytelling, for example, with video and micro-video content, that may be used by scientists and benefit citizens.
— *Microelectronic measurement sensors*. High-performance, pressure, temperature, gas composition and particulate sensors are now made in quantities of tens of millions per year at low cost.
— *Open-source accessible platforms* have led to a library of code and circuits allowing for direct integration of the power of microcontrollers in sensing systems [10].
— *Rapid prototyping* technologies (e.g. parametric CAD tools, laser and water jet cutters, 3D printing and computer numerically controlled (CNC) machining) have slashed the cost and time required to make prototypes and e-sharable designs.
— *Telemetry*. Low-power inexpensive wireless communication is becoming available worldwide, closing the gap between remote and disconnected regions of significant environmental interest (e.g. TAHMO across 23 African countries sends 5 min resolution data for $0.25/month/station).
— *Copyright*. The CERN-OHL-S [28] copyright allows unlimited non-commercial use while maintaining strong incentives for commercial licensing and co-development.
— *Remote collaboration*. A global community can now work seamlessly based on structured project management systems, fully digital cloud-connected design tools and broad access to video conferencing.

### (a) The technological impact of OPEnS

At Open-Sensing.org, one can find documentation of the 28 projects undertaken at OPEnS thus far (a selection of that may be found in table 1). The span of efforts was unexpected. We have been asked to sample methane from cow belches, landslides, tree growth, soil moisture, COVID DNA in sewage, insect mating calls, the isotopic concentration of rainfall and to capture the DNA from flowing water. Many of these have had electromechanical features, but several have been purely mechanical. Many people have asked Selker the focus of his work, and the OPEnS lab has more pointedly answered this question than any soliloquy could provide: our focus is on a problem-solving process. Great questions beget interesting answers, and the OPEnS lab is a venue for people with tough questions in need of new ways they might gain opportunity. The clients know their needs, and OPEnS can assemble a team of engineers that can translate these into detailed specifications, a design process and a set of solutions. Having seen the power of this structured collaborative process, it is astonishing to us that such laboratories are not ubiquitous. We hope funding agencies will take note.

## 3. Looking forward

### (a) Engagement of the commercial sector

A key lesson from OPEnS has been the dual importance of open-source publication and commercialization. We have found that there are few people who wish to build their own instruments, and frankly, it makes sense: homemade tools take vast effort to build and are often not as reliable as commercial products. At the same time, the opportunities for companies to do good business in environmental sensing is huge: ‘The environmental sensors market was valued at USD1.4 billion in 2020, and it is expected to register a CAGR of over 9.25% during the forecast period (2021−2026), to reach a value of USD2.17 billion, by 2026’ [29]. Employing the CERN-OHL-S licensing, OPEnS designs are free and open for non-commercial use. Should a commercial entity choose to produce the design based on CERN-OHL-S copyrighted material, they are obliged to subsequently publish all innovations and alterations of the design. This commercially onerous requirement can be avoided through direct licensing from the creator, allowing companies willing to invest in building protected intellectual property for commercial versions. The OPEnS lab is currently in commercialization discussions with several major environmental sensing companies.

### (b) Machine discoverable data

Sensors are a starting place for improved environmental monitoring, but by no means are sufficient to realize the long-term opportunity. Specifically, we are concerned that most sensor data are lost after the first use. We believe it is critical to address the grand challenge of developing and implementing a ‘Born-FAIR’ (Findable, Accessible, Interoperable and Reusable) data standard to democratize data. In this framework, when launching a sensor, the device employed to initiate operation will survey the installer for a comprehensive metadata context to be permanently affiliated with the measurements from ‘birth’. Such metadata will serve the installing team, but, more significantly, allow the data to be discovered and appropriately employed by future researchers. We urgently need to turn the corner on datasets that serve just one team for one objective by making all Earth observations machine discoverable. We see the next challenge for the community to be the development of widely adopted metadata standards for sensors that will enhance the value of all sensors.

### (c) Moving from pilot to scale

After nearly a decade of operation, it is clear the OPEnS model can be impactful both to science and education. We see a great opportunity for this model to be expanded at other universities and in other disciplines. The OPEnS lab has always sought to be a global collaborative enterprise, and OPEnS has been engaged across the US and around the world. We are now actively pursuing the establishment of ‘sister’ OPEnS labs that can build on our experience and critical mass. We see that a key barrier to the establishment of new sites is the issue of critical mass: space, equipment, expertise, connection to clients and base funding must all be in place. How can we lower this barrier? The OPEnS lab has had visiting scientists from Germany, Israel [30], Taiwan, the Netherlands, Ghana, Japan, Switzerland and across the US. These visits have generally resulted in one new design which the departing team can continue to advance, often with continuing input from OPEnS. Our students gain huge benefits from travelling to remote labs where they take on the role of experts. We believe that sister labs will build from these efforts, typically beginning with a single line of work and then expanding as opportunities emerge. We are currently seeking funding to support this expansion of the OPEnS model and welcome partners of all kinds, from presenting a need for a new sensor to a desire to establish a new facility that draws upon the OPEnS experience.

## Data Availability

This article has no additional data.
